# Expression patterns of bone morphogenetic protein 7 (BMP7) and its prognostic roles in neuroblastoma: An integrated bioinformatics analysis

**DOI:** 10.1371/journal.pone.0340718

**Published:** 2026-02-03

**Authors:** Haiwei Wang, Xinrui Wang, Liangpu Xu

**Affiliations:** Medical Genetic Diagnosis and Therapy Center, Fujian Key Laboratory for Prenatal Diagnosis and Birth Defect, Fujian Maternity and Child Health Hospital, Affiliated Hospital of Fujian Medical University, Fuzhou, Fujian, China; Goethe University Hospital Frankfurt, GERMANY

## Abstract

**Background:**

Bone morphogenetic proteins (BMPs) are associated with the prognosis of various types of adult cancers. However, the expressions and prognosis of BMPs in pediatric neuroblastoma remain unclear.

**Methods:**

Six publicly available neuroblastoma cohorts were downloaded for bioinformatics analysis. The prognosis of BMPs in neuroblastoma was determined using cox regression analysis and Kaplan-Meier survival analysis.

**Results:**

Our study revealed that, compared to other BMP family members, BMP1, BMP7 and BMP8B were highly expressed in neuroblastoma. However, only BMP7 was associated with the prognosis of neuroblastoma in all six neuroblastoma cohorts. Higher BMP7 expression was associated with the shorted event free survival and overall survival of neuroblastoma. The prognosis of BMP7 in neuroblastoma was independent of age and MYCN amplification. The expressions of BMP7 were higher in neuroblastoma patients with MYCN amplification or age ≥ 18months or in stage 4 neuroblastoma. Moreover, higher BMP7 was associated with the shorted event free survival and overall survival of MYCN amplified or stage 4 neuroblastoma. At last, we found that breast cancer metastasis suppressor 1 (BRMS1) was correlated with BMP7 expression. However, in contrast with the suppressor role of BRMS1 in adult cancers, BRMS1 was significantly associated with the unfavorable clinical outcomes of neuroblastoma. Overall, our analysis the demonstrated correlations between BMPs and clinical features of neuroblastoma and provided unique therapeutic targets for neuroblastoma treatments.

**Conclusions:**

BMP7 was a prognostic maker of neuroblastoma.

## Introduction

Bone morphogenetic proteins (BMPs) are secreted cytokines belonging to transforming growth factor β super-family [1]. To date, more than 15 different members of BMPs have been identified [2]. After binding to the receptors, BMPs stimulate various signaling pathways to maintain organ homeostasis by regulating cell proliferation, differentiation and survival [3]. The alterations of BMPs or BMPs signaling pathways could lead to multiple diseases, such as cardiovascular, musculoskeletal diseases and cancers [4]. BMPs antagonists or agonists are promising pharmacological drugs for such diseases [5]. So, comprehending the associations of BMPs and specific clinical features of diseases will provide unique therapeutic alternatives.

Neuroblastoma is a prevalent malignant pediatric disease. The outcomes of high-risk neuroblastoma are still not satisfactory and treatments of high-risk neuroblastoma are limited [6,7]. Recent evidences demonstrate that BMPs play significant roles in the treatments of neuroblastoma. For example, BMP6 and all-trans retinoic acid can synergistically induce the differentiation of neuroblastoma cells [8]. Moreover, BMP2 [9] and BMP4 [10] are involved in the growth inhibition and cell differentiation of neuroblastoma. However, the expressions and prognosis of BMPs in neuroblastoma are unclear. And the functions of other BMPs members in neuroblastoma are not reported. Understanding the functions of BMPs in neuroblastoma will provide mechanistic information which contributes to selective of BMPs antagonists or agonists for neuroblastoma treatments [5].

In this study, we analyzed the expressions and prognosis of 16 BMPs members in 1847 neuroblastoma patients derived from six published neuroblastoma cohorts. Our findings indicate that, in contrast to other BMPs, BMP7 was an independent prognostic marker and was significantly associated with the prognosis of neuroblastoma. We also found that breast cancer metastasis suppressor 1 (BRMS1) was correlated with BMP7 expression and BRMS1 was significantly correlated with the outcomes of neuroblastoma. Overall, our analysis illustrates the relationships of BMPs and clinical features of neuroblastoma and provides insight into potential therapeutic targets for future neuroblastoma treatments.

## Materials and methods

### Data collection and processing

GSE16476 [11–13], GSE62564 [14,15] and GSE85047 [16] were obtained from the Gene Expression Omnibus (GEO) (www.ncbi.nlm.nih.gov/geo). Expression values in GEO microarrays were standardized using Robust Multi-array Average (RMA). Neuroblastoma cohorts in Therapeutically Applicable Research to Generate Effective Treatments (TARGET) were collected from https://ocg.cancer.gov/ [17]. Expression values in TARGET based on RNA-seq method were standardized using log-transformed Fragments Per Kilobase of transcript per Million mapped reads (FPKM). E-MTAB-1781 [18] and E-TABM-38 [19] datasets were downloaded from The Functional Genomics Data Collection (https://www.ebi.ac.uk/arrayexpress/), were also standardized using RMA method. All datasets were processed and annotated using R software, and relative gene expression values were used for further studies. Detailed information of the neuroblastoma cohorts was provided in S1 Table.

### Univariate and multivariable cox regression analysis

We used univariate and multivariable cox regression analysis based on “survival” and “survminer” packages in R software to determine genes associated with the prognosis of neuroblastoma.

### Kaplan-Meier survival analysis

Kaplan-Meier survival analysis based on “survival” and “survminer” packages in R software was used to determine genes associated with the prognosis of neuroblastoma. Based on gene expression values, neuroblastoma patients were classified into two groups using “surv_cutpoint” function from the “survminer” package. “Surv_cutpoint” function evaluates all possible cutpoints for each continuous variable, computes the log-rank statistic at each potential cutpoint and selects the cutpoint that maximizes the separation between survival curves.

### Correlation analysis

Spearman correlations of BMP7 and BRMS1 were carried out using R software “lm” method and “corrplot” package in neuroblastoma cohorts.

### Statistical analysis

Statistical difference of neuroblastoma sub-groups was performed using the two tails un-paired student’s t test. Cox regression survival analysis determined the p-values of the BMPs prognosis. P values in Kaplan-Meier survival analysis were determined by log-rank test. P value < 0.05 was chosen to be significantly different.

## Results

### Expressions and prognosis of BMPs in neuroblastoma

**Fig 1:**
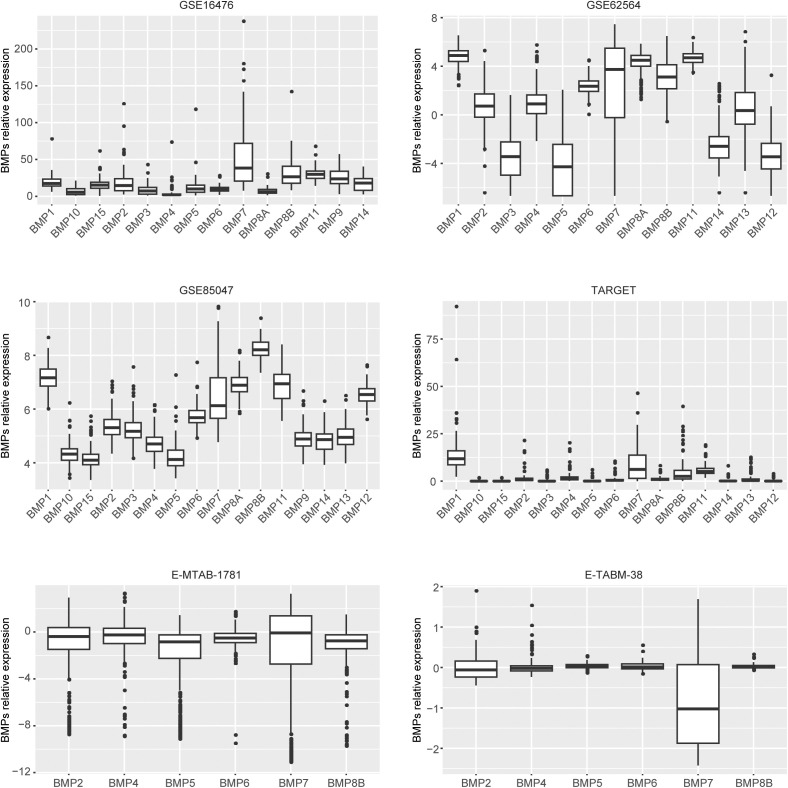
Expressions of BMPs in neuroblastoma. Relative expressions of BMPs in GSE16476, GSE62564, GSE85047, TARGET, E-MTAB-1781 and E-TABM-38 neuroblastoma cohorts.

To analyze the expressions and prognosis of BMPs in neuroblastoma, transcriptional profiling of 1847 neuroblastoma patients derived from six published neuroblastoma cohorts were collected (S1 Table). And 16 members of BMPs were studied, including BMP1–7, BMP8A, BMP8B, GDF2 (BMP9), BMP10, GDF11 (BMP11), GDF7 (BMP12), GDF6 (BMP13), GDF5 (BMP14) and BMP15. The expressions of BMPs in neuroblastoma were found to be significantly varied. BMP1, BMP7 and BMP8B were relatively highly expressed in neuroblastoma in GSE16476, GSE62564, GSE85047 and TARGET datasets (Fig 1). However, other BMPs were barely detected in those datasets (Fig 1). Only a few BMPs were detected in E-MTAB-1781 and E-TABM-38 datasets. Nonetheless, the expressions of BMPs in E-MTAB-1781 and E-TABM-38 neuroblastoma were not significantly different (Fig 1).

Moreover, the prognosis of BMPs in neuroblastoma was determined using univariate cox regression analysis. Our findings showed that BMP1, BMP6, BMP7 and BMP9 were associated with the event free survival of neuroblastoma In GSE16476 dataset (S1 Fig). Similarly, BMP1, BMP6, BMP7, BMP8A and BMP8B were prognostic factors of event free survival in GSE62564 dataset (S1 Fig). In addition, BMP7 was associated with the event free survival of neuroblastoma in GSE85047 and TARGET datasets, as well as in E-MTAB-1781 and E-TABM-38 datasets where only a few BMPs were detected (S1 Fig).

**Fig 2:**
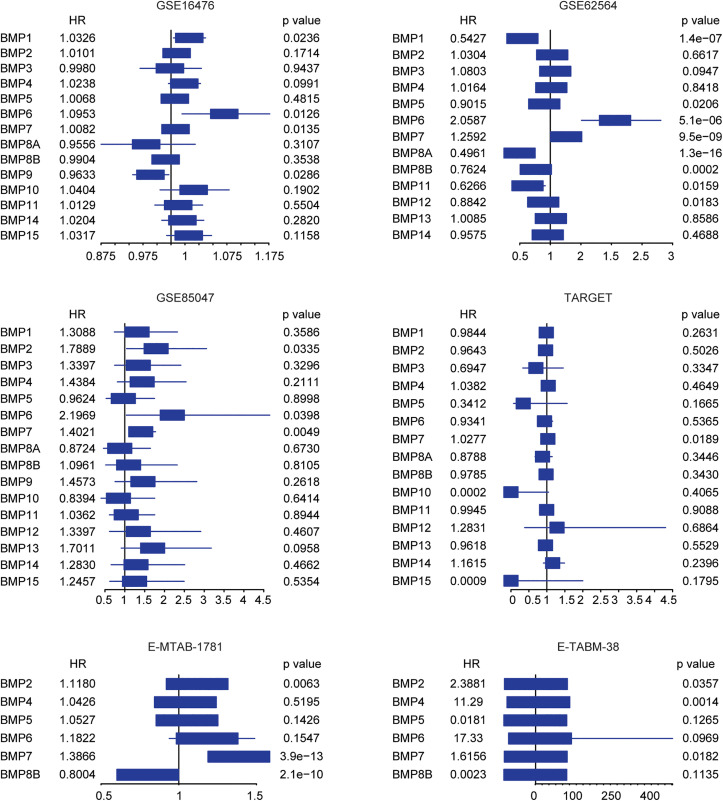
Prognosis of BMPs in neuroblastoma. Forest plots showed the associations of BMPs expressions with the neuroblastoma overall survival in six cohorts. Hazard ratio (HR) and p values were determined by univariate cox regression analysis.

Furthermore, BMP1, BMP6, BMP7 and BMP9 were associated with the overall survival of neuroblastoma in GSE16476 dataset (Fig 2). Similarly, BMP1, BMP5, BMP6, BMP7, BMP8A, BMP8B, BMP11 and BMP12 were associated with the overall survival of neuroblastoma in GSE62564 dataset (Fig 2). BMP7 was associated with the overall survival of neuroblastoma in GSE85047, TARGET, E-MTAB-1781 and E-TABM-38 datasets (Fig 2). Results from the six independent neuroblastoma cohorts suggested that BMP7 exhibited high expression and was associated with the event free survival and overall survival of neuroblastoma.

### High expression levels of BMP7 are associated with the unfavorable prognosis of neuroblastoma

**Fig 3:**
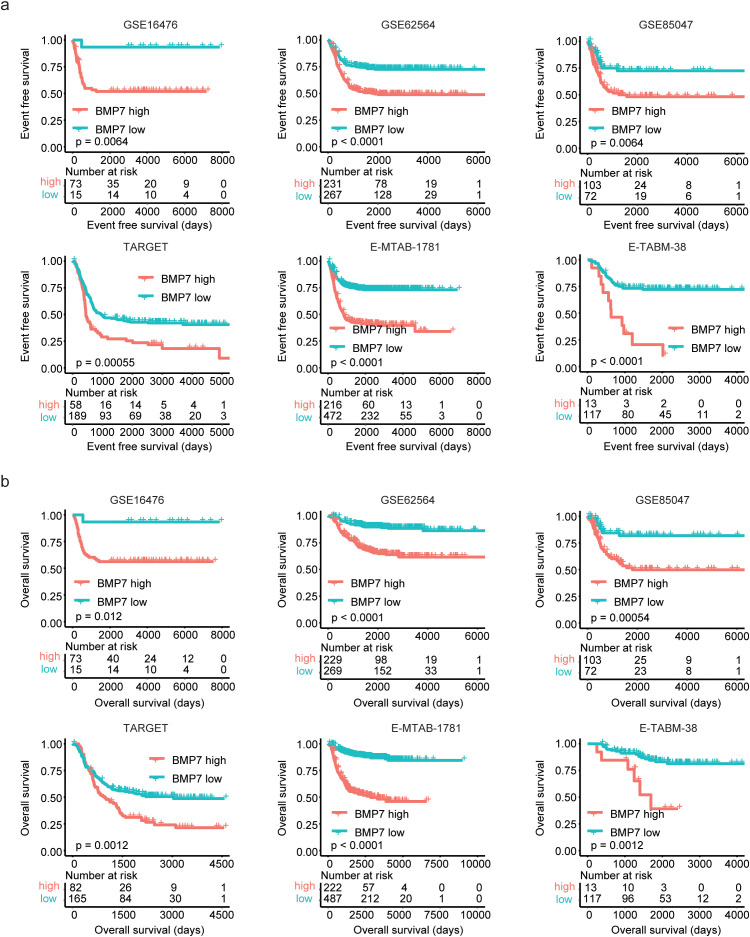
High expression levels of BMP7 are associated with the unfavorable prognosis of neuroblastoma. (a) The Kaplan-Meier curves showed the event free survival of neuroblastoma patients with BMP7 higher expressions or lower expressions in six cohorts. (b) Overall survival of neuroblastoma patients with BMP7 higher expressions or lower expressions.

BMP7 also known as osteogenic protein 1, plays essential roles in bone, kidney and brown adipose development. However, relationships between BMP7 and neuroblastoma prognosis have never been reported. Kaplan-Meier survival analysis was used to further investigate the prognosis of BMP7 in neuroblastoma. Using optimal cutoff points based on BMP7 expression values, neuroblastoma patients were classified into BMP7 high and BMP7 low expression groups. The results revealed that neuroblastoma patients with lower BMP7 expressions had a significantly longer event-free survival compared to patients with higher BMP7 expression levels in GSE16476, GSE62564, GSE85047, TARGET, E-MTAB-1781 and E-TABM-38 neuroblastoma cohorts (Fig 3a). Additionally, neuroblastoma patients with lower BMP7 expression levels were associated with prolonged overall survival compared to those with higher BMP7 expression levels in the aforementioned datasets (Fig 3b).

### BMP7 is an independent prognostic factor of neuroblastoma

**Fig 4:**
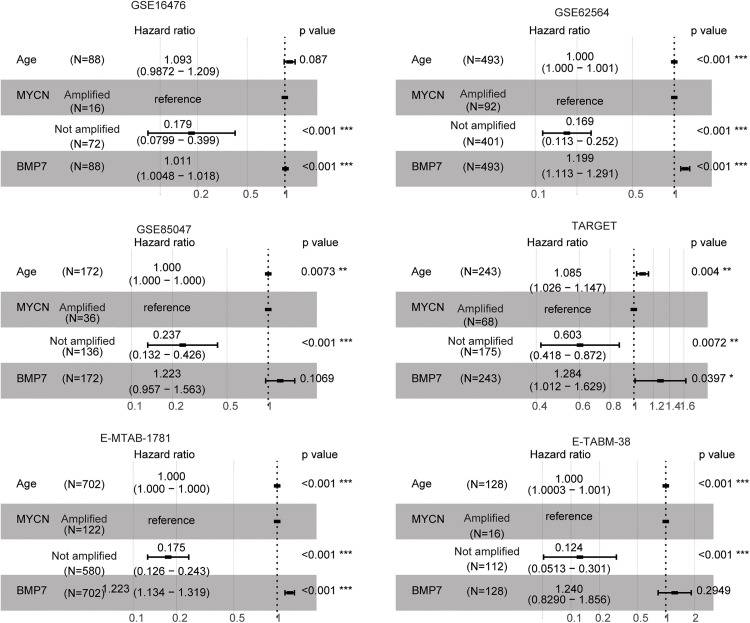
BMP7 is an independent prognostic factor of neuroblastoma. Forest plots showed the associations of age, MYCN amplification and BMP7 with the overall survival of neuroblastoma. Hazard ratio and p values were determined by multivariate cox regression assay.

Age, MYCN amplification and stage are recognized prognostic markers of neuroblastoma [20]. Multivariate cox regression analysis was employed to explore the relationship between these markers and BMP7 expression in predicting overall survival of neuroblastoma. Age was an independent prognostic factor in GSE62564, GSE85047, TARGET, E-MTAB-1781 and E-TABM-38 datasets, while not in GSE16476 dataset (Fig 4). Similarly, MYCN amplification was found to be an independent prognostic factor in GSE16476, GSE62564, GSE85047, TARGET, E-MTAB-1781 and E-TABM-38 datasets (Fig 4). On the other hand, BMP7 was identified as an independent prognostic factor in GSE16476, GSE62564, TARGET and E-MTAB-1781 neuroblastoma cohorts (Fig 4). However, BMP7 was not an independent prognostic factor of neuroblastoma in GSE85047 and E-TABM-38 datasets (Fig 4).

### Expressions and prognosis of BMP7 in MYCN amplified or non-amplified neuroblastoma

**Fig 5:**
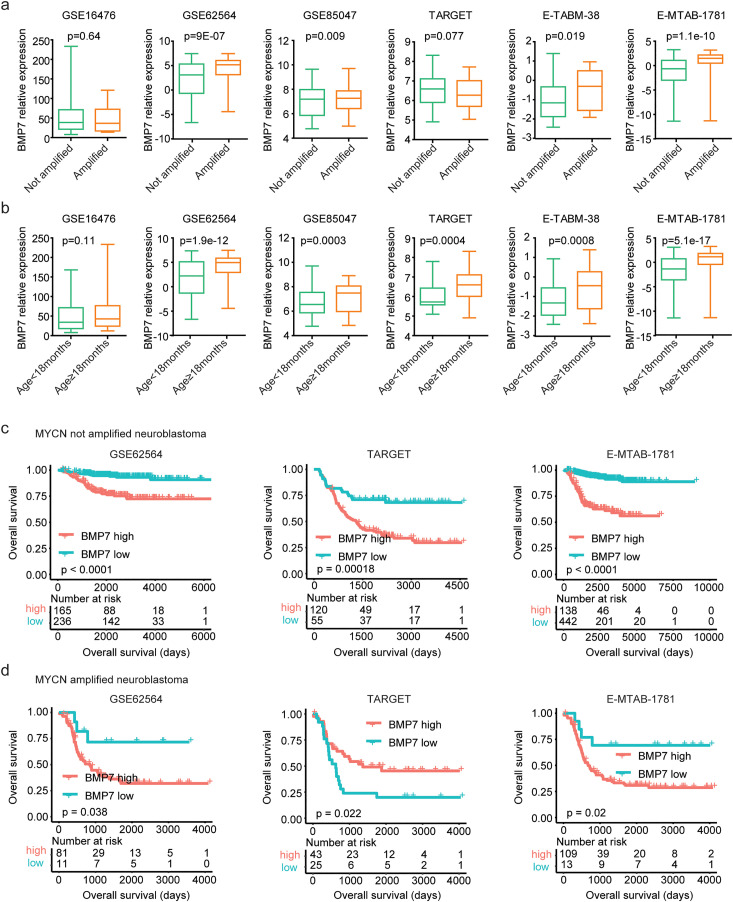
Expressions and prognosis of BMP7 in MYCN amplified or non-amplified neuroblastoma. (a) Box plots showed the relative BMP7 expression levels in neuroblastoma patients with or without MYCN amplification. P values were performed using two tails un-paired student’s t test. (b) The relative BMP7 expression levels in neuroblastoma patients with age ≥ 18month or <18months. (c) The Kaplan-Meier curves showed the overall survival of MYCN non-amplified neuroblastoma patients with BMP7 higher expressions or lower expressions in GSE62564, TARGET and E-MTAB-1781. (d) Overall survival of MYCN amplified neuroblastoma patients with BMP7 higher expressions or lower expressions in GSE62564, TARGET and E-MTAB-1781.

Neuroblastoma exhibits distinct sub-types because of the different clinical features. We determined the BMP7 expressions in different sub-types of neuroblastoma in six independent neuroblastoma cohorts. BMP7 was up-regulated in neuroblastoma patients with MYCN amplification, as demonstrated by the GSE62564, GSE85047, E-MTAB-1781 and E-TABM-38 cohorts (Fig 5a). Additionally, the expression levels of BMP7 were higher in neuroblastoma patients with age ≥ 18months than neuroblastoma patients with age < 18months in GSE62564, GSE85047, TARGET, E-MTAB-1781 and E-TABM-38 cohorts (Fig 5b). However, BMP7 expressions were not associated with age or MYCN amplification in GSE16476 dataset (Fig 5a, b).

Prognosis of BMP7 in MYCN amplified or non-amplified sub-types of neuroblastoma was further studied. MYCN non-amplified neuroblastoma patients with BMP7 higher expression levels had shorted event free survival in GSE62564, TARGET and E-MTAB-1781 datasets (S2 Figa). In MYCN amplified neuroblastoma cohorts, BMP7 higher expression levels were significantly associated with shorted event free survival in TARGET and E-MTAB-1781 datasets, but not significant in GSE62564 dataset (S2 Figb).

Moreover, MYCN non-amplified neuroblastoma patients with BMP7 higher expression levels had shorted overall survival in GSE62564, TARGET and E-MTAB-1781 neuroblastoma cohorts (Fig 5c). Also, in MYCN amplified neuroblastoma cohorts, BMP7 higher expression levels had shorted overall free survival in GSE62564 and E-MTAB-1781, but not in TARGET dataset (Fig 5d). Hence, BMP7 could serve as a prognostic marker in both MYCN amplified and non-amplified neuroblastoma sub-types.

### Expressions and prognosis of BMP7 in stage 4 neuroblastoma

**Fig 6:**
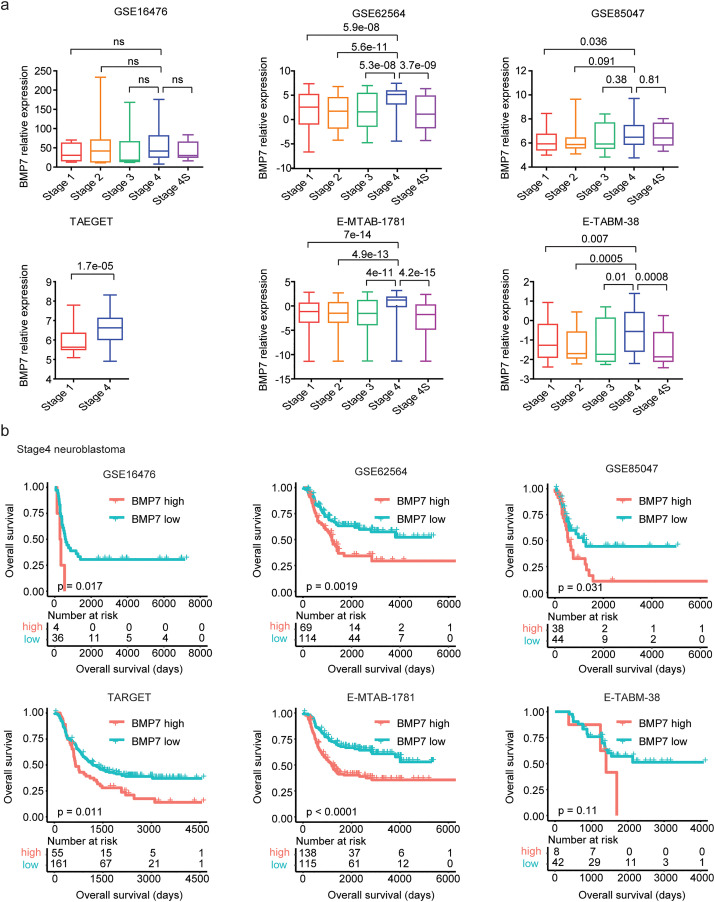
Expressions and prognosis of BMP7 in stage 4 neuroblastoma. (a) The relative BMP7 expression levels in neuroblastoma patients with different stages. (b) The Kaplan-Meier curves showed the different overall survival of stage 4 neuroblastoma patients with BMP7 higher expressions or lower expressions.

Furthermore, contrast with stage 1, stage 2, stage 3 and stage 4s neuroblastoma, BMP7 was up-regulated in stage 4 neuroblastoma in GSE62564, E-MTAB-1781 and E-TABM-38 cohorts (Fig 6a). We also observed an increase in BMP7 expression in stage 4 neuroblastoma patients compared to stage 1 neuroblastoma patients in the GSE85047 and TARGET datasets (Fig 6a). However, in the GSE16476 dataset, no significant difference was found in BMP7 expression levels between the different stages of neuroblastoma (Fig 6a).

Prognosis of BMP7 in stage 4 neuroblastoma were further studied. Stage 4 neuroblastoma patients with BMP7 higher expression levels had significantly shorted event free survival than those with low levels of BMP7 expressions in GSE16476, GSE62564, TARGET, E-MTAB-1781 and E-TABM-38 cohorts, while not in GSE85047 dataset (S3 Fig). Also, Stage 4 neuroblastoma patients with BMP7 higher expression levels had shorted overall survival compared to those with low levels of BMP7 expressions in GSE16476, GSE62564, GSE85047, TARGET and E-MTAB-1781 datasets, but not in E-TABM-38 dataset (Fig 6b).

### Identification of genes associated with BMP7

**Fig 7:**
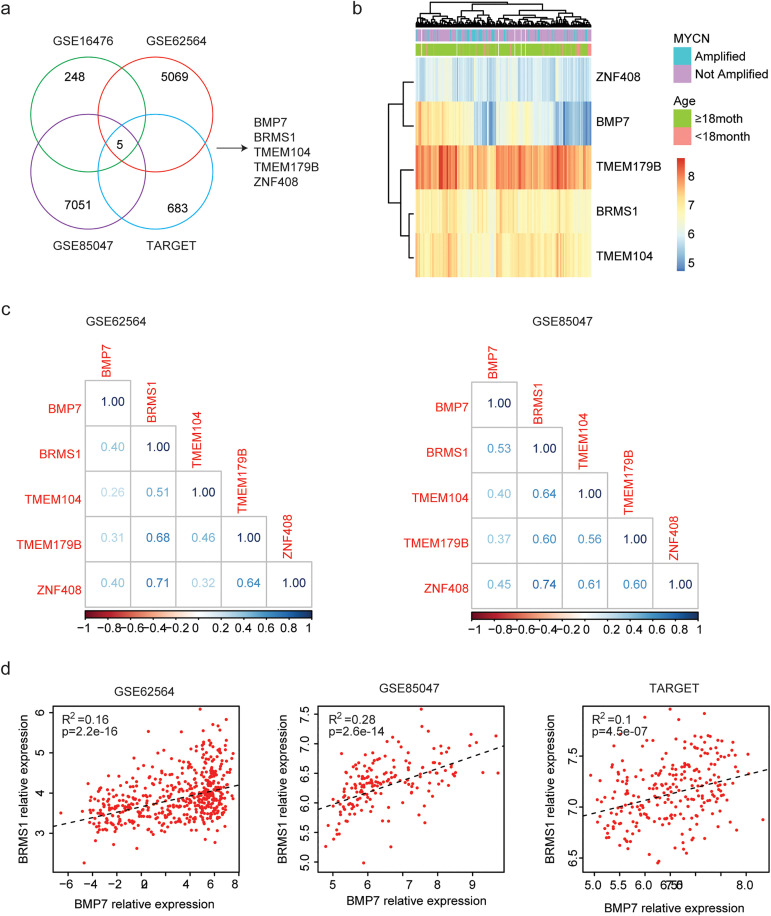
Identification of genes associated with BMP7 expressions. (a) Overlapped genes associated with BMP7 expressions in GSE16476, GSE62564, GSE85047 and TARGET datasets. (b) Clustering heatmaps showed the genes associated with BMP7 expressions in TARGET dataset. (c) Correlations of BMP7 with BRMS1, TMEM179B, TEME104 and ZNF408 in GSE62564 and GSE85047 datasets. (d) Correlations of BMP7 and BRMS1 in GSE62564, GSE85047 and TARGET datasets.

To further understand the prognostic effects of BMP7, genes associated with BMP7 were studied in neuroblastoma. In comparison to neuroblastoma patients with lower BMP7 expressions, neuroblastoma patients with higher BMP7 expressions in GSE16476, GSE62546, GSE85047, and TARGET datasets had 248, 5069, 7051, and 683 changed genes, respectively (Fig 7a). Additionally, five genes were commonly associated with BMP7 expressions (Fig 7a). The expression levels of these five genes were further demonstrated in TARGET dataset. TMEM179B, BRMS1 and TEME104 were relatively highly expressed in neuroblastoma, while, ZNF408 was expressed at low levels in neuroblastoma (Fig 7b).

Breast cancer metastasis suppressor 1 (BRMS1) is first identified as a suppressor of breast cancer metastasis and is involved in the progression of multiple types of adult cancers. However, the expressions of BRMS1 in neuroblastoma have never been reported. We found that, compared with TMEM179B TEME104 and ZNF408, BRMS1 was more significantly correlated with BMP7 expression in GSE85047 dataset (Fig 7c). Moreover, in GSE62546, GSE85047 and TARGET datasets, neuroblastoma patients with higher BMP7 expressions were also with higher BRMS1 expressions (Fig 7d), suggesting that BRMS1 could be an unfavorable prognostic factor of neuroblastoma.

### High expression levels of BRMS1 are associated with the unfavorable prognosis of neuroblastoma

**Fig 8:**
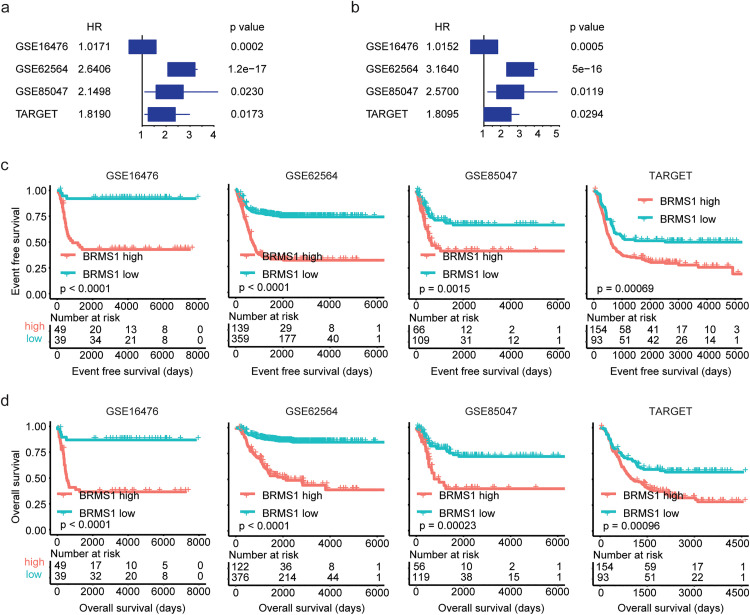
High expression levels of BRMS1 are associated with the unfavorable prognosis of neuroblastoma. (a) Forest plots showed the associations of BRMS1 expressions with the neuroblastoma event free survival in GSE16476, GSE62564, GSE85047 and TARGET cohorts. (b) Forest plots showed the associations of BRMS1 expressions with the neuroblastoma overall survival. (c) The Kaplan-Meier curves showed the event free survival of neuroblastoma patients with BRMS1 higher expressions or lower expressions in GSE16476, GSE62564, GSE85047 and TARGET cohorts. (d) Overall survival of neuroblastoma patients with BRMS1 higher expressions or lower expressions.

The univariate cox regression analysis was further used to determine the unfavorable prognosis of BRMS1 in neuroblastoma. In GSE16476, GSE62564, GSE85047 and TARGET datasets, BRMS1 was significantly correlated with the event free survival of neuroblastoma (Fig 8a). Additionally, BRMS1 was significantly associated with the overall survival of neuroblastoma in these same datasets (Fig 8b).

Kaplan-Meier survival analysis was also used to validate the prognostic impact of BRMS1 on neuroblastoma. Consistent with the findings of the univariate cox regression analysis, neuroblastoma patients with BRMS1 higher expression levels had shortened event free survival (Fig 8c) and overall survival (Fig 8d), in contrast with BRMS1 lowly expressed neuroblastoma patients in GSE16476, GSE62564, GSE85047 and TARGET cohorts.

## Discussion

Previous reports have demonstrated that BMP7 is highly expressed in tumors and is implicated in the uncontrolled tumor cell proliferation [21]. Furthermore, increased expression of BMP7 has been correlated with poor prognosis of breast cancer [22], colon cancer [23] and lung cancer [24,25]. However, BMP7 expression is down-regulated in kidney cancer [26] and has been shown to inhibit the proliferation and invasion of glioblastoma cells by inducing glioblastoma differentiation [27]. BMP7 administration has also been found to reverse chemo-resistance in colon cancer [28]. Those findings underscore the complex functions of BMP7 in different types of cancers. In this study, we showed that BMP7 was highly expressed in neuroblastoma and higher expression of BMP7 was associated with the unfavorable prognosis of neuroblastoma. Therefore, we hypothesize that neuroblastoma patients may benefit from BMP7 inhibition due to the excessive BMP7 activity in these tumors.

Neuroblastoma is a heterogeneous disease and its progression varies significantly [29]. Age, MYCN amplification and stages are prognostic biomarkers of neuroblastoma [20]. Our results showed that BMP7 was a prognostic factor of neuroblastoma, regardless of age and MYCN amplification. The expression levels of BMP7 were higher in neuroblastoma patients with MYCN amplification or age ≥ 18months or in stage 4 neuroblastoma. Moreover, higher BMP7 was associated with the shorted event free survival and overall survival in MYCN amplified or stage 4 neuroblastoma. Those results suggested BMP7 was also associated with the prognosis of high-risk of neuroblastoma. And high-risk neuroblastoma patients may be also benefit from BMP7 inhibition.

BRMS1 is a transcriptional suppressor and is first identified as suppressing the metastasis but not the growth, of human breast cancer cells [30]. Moreover, BRMS1 dramatically suppresses the metastatic potential of melanoma [31,32], non-small cell lung cancer [33] and ovarian cancer [34]. However, no available articles address the relationships between BRMS1 expression and clinical outcomes of neuroblastoma. In this study, we found that BRMS1 expression was correlated with BMP7 and higher BRMS1 expression was significantly associated with the worse outcomes of neuroblastoma suggesting the tumor promoting roles of BRMS1 in neuroblastoma. Therefore, our analysis revealed different functions of BRMS1 and suggested that inhibitions of BRMS1 were selective strategies for neuroblastoma treatments.

To the best of our knowledge, this is the first bioinformatics analysis of the roles of BMPs in neuroblastoma. Our results highlighted that BMP7 and BRMS1 were prognostic biomarkers and therapeutic targets for neuroblastoma. However, further validation through neuroblastoma cells is needed to determine whether inhibitions of BMP7 or BRMS1 could induce the suppression of neuroblastoma.

## Conclusions

Contrast with other BMPs, BMP7 was a more significant independent prognostic marker and was significantly associated with the clinical outcomes of high-risk neuroblastoma.

## Supporting information

S1 Table Information about the six published neuroblastoma cohorts used in this study. (DOCX)

S1 Fig Prognosis of BMPs in neuroblastoma. Forest plots showed the associations of BMPs expressions with the neuroblastoma event free survival in GSE16476, GSE62564, GSE85047, TARGET, E-MTAB-1781 and E-TABM-38 cohorts.
(TIF)

S2 Fig Prognosis of BMP7 in MYCN amplified or non-amplified neuroblastoma. (a) The Kaplan-Meier curves showed the event free survival of MYCN non-amplified neuroblastoma patients with BMP7 higher expressions or lower expressions in GSE62564, TARGET and E-MTAB-1781 cohorts. (b) Event free survival of MYCN amplified neuroblastoma patients with BMP7 higher expressions or lower expressions in GSE62564, TARGET and E-MTAB-1781 cohorts.
(TIF)

S3 Fig Prognosis of BMP7 in stage 4 neuroblastoma. The Kaplan-Meier curves showed the event free survival of stage 4 neuroblastoma patients with BMP7 higher expressions or lower expressions in GSE16476, GSE62564, GSE85047, TARGET, E-MTAB-1781 and E-TABM-38 cohorts.
(TIF)

## Abbreviations

BMPs: Bone morphogenetic proteins
BRMS1: Breast cancer metastasis suppressor 1
GEO: Gene Expression Omnibus
TARGET: Therapeutically Applicable Research to Generate Effective Treatments
RMA: Robust Multi-array Average
FPKM: Fragments Per Kilobase of transcript per Million mapped reads
