# Supplementary material for: Expression patterns of bone morphogenetic protein 7 (BMP7) and its prognostic roles in neuroblastoma: An integrated bioinformatics analysis
Source: PLoS One. 2026 Feb 3;21(2):e0340718. doi: 10.1371/journal.pone.0340718 (PMC12867232; doi:10.1371/journal.pone.0340718)
Supplement: S1 Table — (DOCX) [file pone.0340718.s001.docx]

S1 Table. Information about the six published neuroblastoma cohorts used in this study.

| Dataset | Patient number | MYCN non-amplified/amplified | Age <18months/ ≥18month |
| --- | --- | --- | --- |
| GSE16476 | 88 | 72/16 | 57/31 |
| GSE62564 | 498 | 401/92 | 297/196 |
| GSE85047 | 175 | 136/36 | 96/79 |
| TARGET | 247 | 175/68 | 32/215 |
| E-MTAB-1781 | 709 | 580/122 | 421/181 |
| E-TABM-38 | 130 | 112/16 | 74/54 |
