# Supplementary figures and images for: Expression patterns of bone morphogenetic protein 7 (BMP7) and its prognostic roles in neuroblastoma: An integrated bioinformatics analysis
Source: PLoS One. 2026 Feb 3;21(2):e0340718. doi: 10.1371/journal.pone.0340718 (PMC12867232; doi:10.1371/journal.pone.0340718)

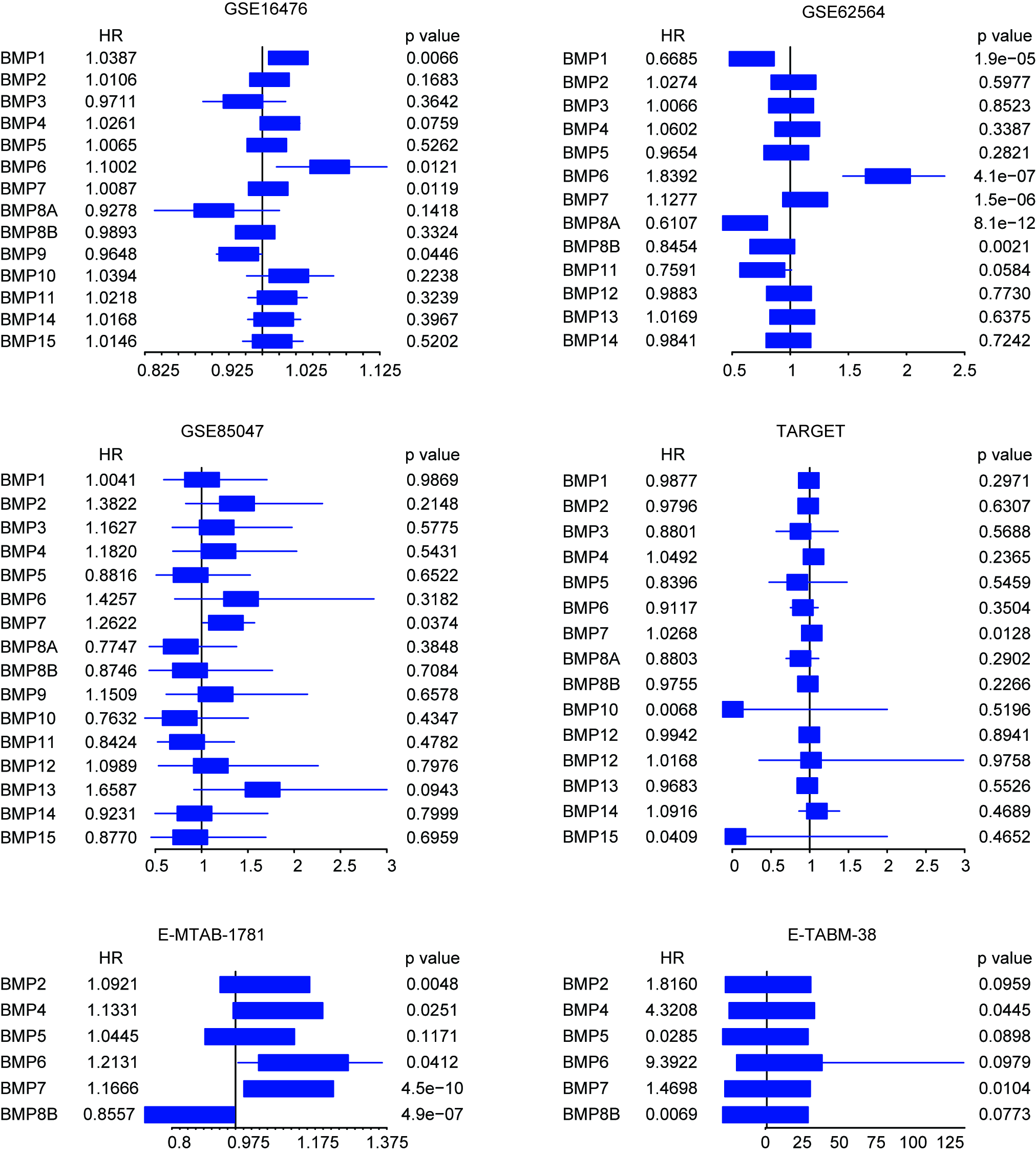

Supplement: S1 Fig — Forest plots showed the associations of BMPs expressions with the neuroblastoma event free survival in GSE16476, GSE62564, GSE85047, TARGET, E-MTAB-1781 and E-TABM-38 cohorts. (TIF) [file pone.0340718.s002.tif]

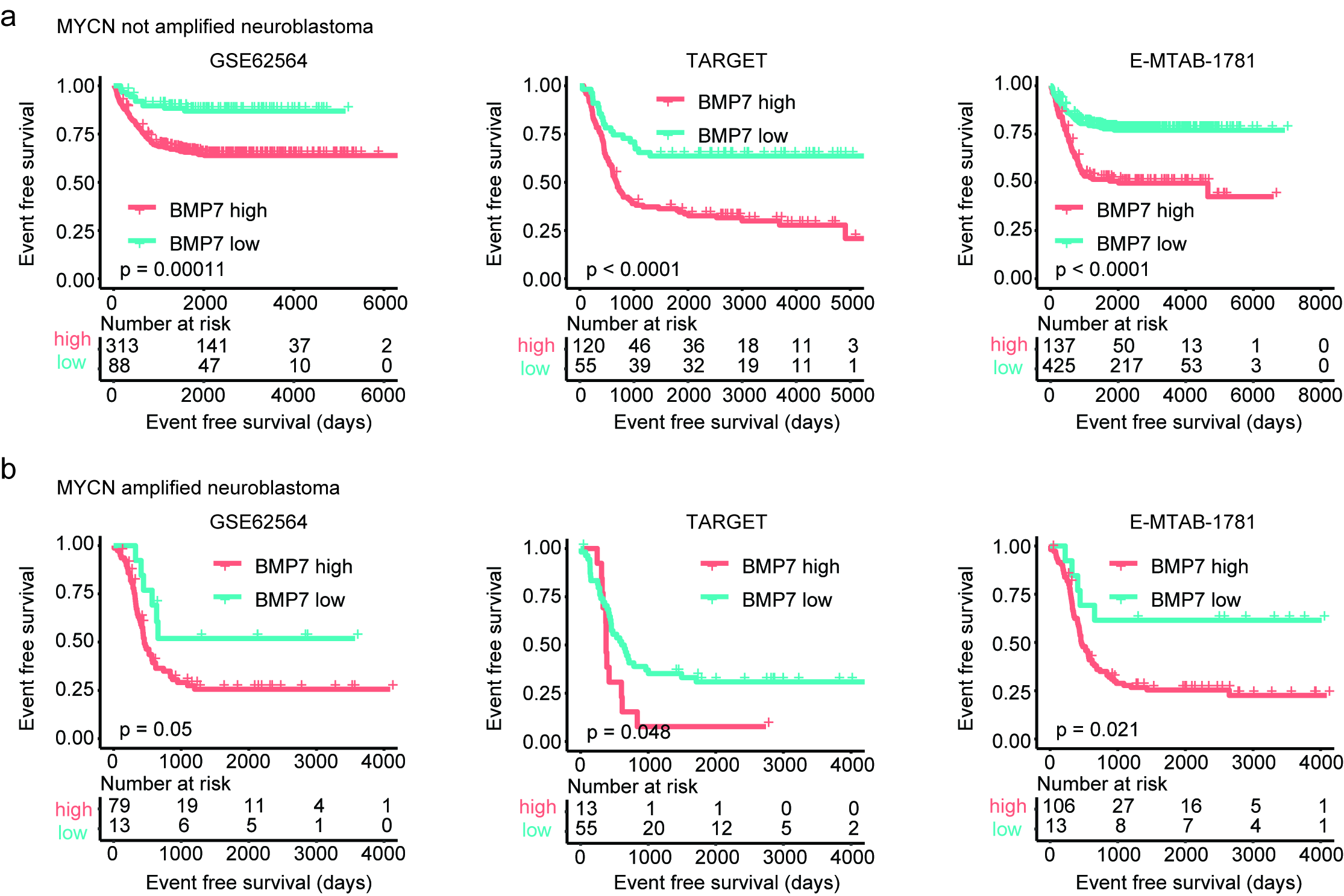

Supplement: S2 Fig — (a) The Kaplan-Meier curves showed the event free survival of MYCN non-amplified neuroblastoma patients with BMP7 higher expressions or lower expressions in GSE62564, TARGET and E-MTAB-1781 cohorts. (b) Event free survival of MYCN amplified neuroblastoma patients with BMP7 higher expressions or lower expressions in GSE62564, TARGET and E-MTAB-1781 cohorts. (TIF) [file pone.0340718.s003.tif]

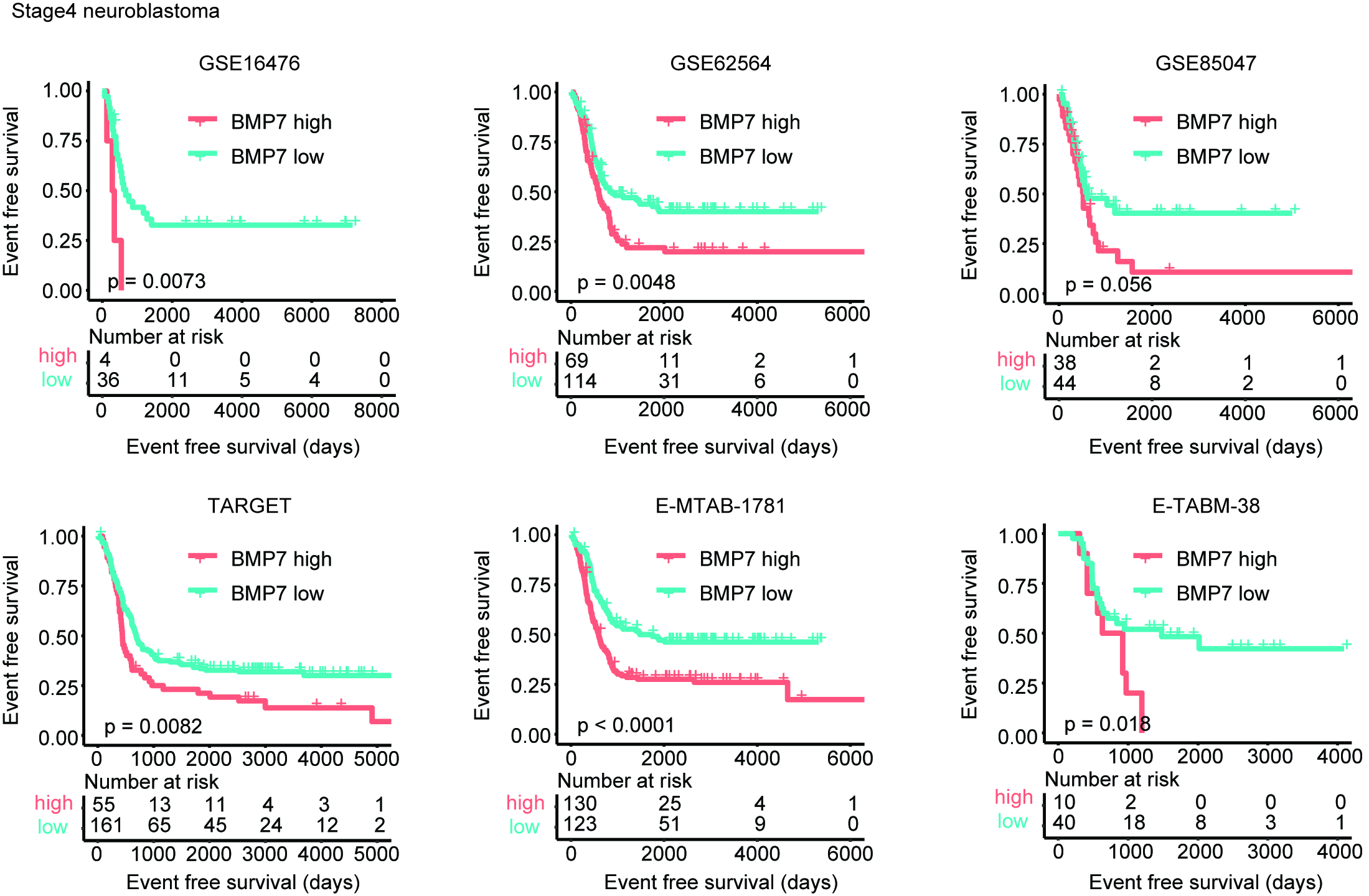

Supplement: S3 Fig — The Kaplan-Meier curves showed the event free survival of stage 4 neuroblastoma patients with BMP7 higher expressions or lower expressions in GSE16476, GSE62564, GSE85047, TARGET, E-MTAB-1781 and E-TABM-38 cohorts. (TIF) [file pone.0340718.s004.tif]
